# Cost-effectiveness of oxaliplatin in the adjuvant treatment of colon cancer in Canada

**DOI:** 10.3747/co.v17i1.436

**Published:** 2010-02

**Authors:** C.L. Attard, J.A. Maroun, K. Alloul, D.T. Grima, L.M. Bernard

**Affiliations:** * Cornerstone Research Group Inc., Burlington, ON; † Ottawa Hospital Regional Cancer Centre, Ottawa, ON; ‡ Sanofi–Aventis Canada Inc., Laval, QC

**Keywords:** Oxaliplatin, cost-effectiveness, adjuvant, chemotherapy, colon cancer, 5fu/lv

## Abstract

**Objective:**

The cost-effectiveness of oxaliplatin in combination with 5-fluorouracil/leucovorin (5fu/lv)—the folfox regimen—was compared with that of 5fu/lv alone as adjuvant therapy for patients with stage iii colon cancer, from the perspective of the Cancer Care Ontario New Drug Funding Program. In the mosaic (Multicenter International Study of Oxaliplatin/5-Fluorouracil/Leucovorin in the Adjuvant Treatment of Colon Cancer) trial, the folfox regimen significantly improved disease-free survival. The mosaic trial formed the basis of the present analysis.

**Methodology:**

Extrapolated patient-level data from the mosaic trial were used to model patient outcomes from treatment until death. Utilities were obtained from the literature. Resource utilization data were derived from the mosaic trial and supplemented with data from the literature. Unit costs were obtained from the Ontario Ministry of Health and Long-Term Care, the London Health Sciences Centre, and the literature.

**Results:**

Lifetime incremental cost-effectiveness ratios for folfox compared with 5fu/lv were CA$14,266 per disease-free year, CA$23,598 per life-year saved, and CA$24,104 per quality adjusted life-year (qaly) gained, discounting costs and outcomes at 5% per annum. These results were stable for a wide range of inputs; only utility values associated with relapse seemed to influence the cost-effectiveness ratios observed.

**Conclusions:**

With an incremental cost of CA$24,104 per qaly gained, folfox is a cost-effective adjuvant treatment for stage iii colon cancer. Compared with 5fu/lv alone, this regimen offers better clinical outcomes and provides good value for money.

## 1. INTRODUCTION

Colorectal cancer includes cancers of both the colon and the rectum, and is the most common form of gastrointestinal cancer. In Canada, colorectal cancer is the fourth most common cancer in terms of incidence rates, both sexes combined, and second only to lung cancer as a cause of cancer death 1. With estimated age-standardized incidence rates for 2009 of 62 and 41 per 100,000 for men and women respectively, colorectal cancer is expected to develop in 1 in every 14 men and 1 in every 16 women during their lifetime, with 1 in 27 men and 1 in 31 women dying from the disease 1. In 2009, an estimated 22,000 Canadians will be diagnosed with colorectal cancer, and 9100 will die 1. A recent Canadian analysis 2 noted that any modest decreases in incidence and mortality rates are more than offset by the increasing numbers of new cases attributable to the aging population. There is thus no sign of the burden of colorectal cancer abating. With so many at risk, effective treatments for colon cancer are critical.

In 2007, based on results of the mosaic (Multi-center International Study of Oxaliplatin/5-Fluorouracil/Leucovorin in the Adjuvant Treatment of Colon Cancer) study, Health Canada approved oxaliplatin (Eloxatin: Sanofi–Aventis Canada Inc., Laval, QC) in combination with 5-fluorouracil/leucovorin (5fu/lv) as adjuvant treatment in patients with stage iii colon cancer.

In mosaic, a large international randomized phase iii trial (146 centres), the folfox4 regimen (oxaliplatin/5fu/lv, hereinafter “folfox”) was compared with 5fu/lv alone in patients with stage ii or iii colon cancer 3. At a median follow-up of 3 years, the addition of oxaliplatin reduced the risk of recurrence by 23% in stage ii and iii patients who had undergone surgery for their primary tumour and by 24% in the subset of stage iii patients. After 77 months of follow-up, this benefit in stage iii patients was confirmed at the 5-year mark (22% risk reduction in relapse or disease recurrence) 4. At a median follow-up of 6 years, a 20% reduction in risk of death was observed in stage iii colon cancer patients 5. Furthermore, as of the January 2007 cut-off update, significant benefits in overall survival (os) were also seen in the intention-to-treat (itt) population 5. These results support folfox as the current standard for adjuvant therapy in colon cancer 6. In fact, based on a review of colon cancer patient charts conducted for cases resected in 2007–2008 for Cancer Care Ontario (cco), folfox was the chemotherapy agent most commonly used in Ontario, with 78.6% of patients treated outside of clinical trials receiving this regimen 7.

Currently, folfox is reimbursed for stage iii colon cancer in all Canadian provinces. Ontario and British Columbia further reimburse it in high-risk stage ii colon cancer and in rectal cancer. Decisions to fund are based on not only the clinical benefit of a product, but also whether the product provides reasonable value in terms of clinical benefit for the cost of treatment. The cost–utility analysis (cua) presented here was conducted to determine the value—in terms of the incremental costs per life-year (ly) gained and per quality-adjusted life-year (qaly) gained—of oxaliplatin as used in the folfox regimen, compared with infusional 5fu/lv alone, for the adjuvant therapy of patients with completely resected stage iii (Dukes C) colon cancer. In October 2008 in Ontario, the recommendations of the Committee to Evaluate Drugs to fund oxaliplatin were based, in part, on the results reported here 6.

## 2. METHODS

### 2.1 Study Design

The cua compared oxaliplatin in combination with 5fu/lv administered per the folfox4 regimen against 5fu/lv alone, using data at the level of the individual patient from the previously reported mosaic trial 3. The mosaic phase iii trial included 2246 patients with stage ii (40%) and iii (60%) colon cancer whose tumours had been completely surgically removed. The primary trial endpoint was disease-free survival (dfs) at 3 years. Secondary trial endpoints included toxicity and os. Outcomes were analyzed based on the itt population. Patients were randomized to receive 5fu/lv or folfox every 2 weeks for 12 cycles as defined in the mosaic trial and recommended by cco guidelines 8.

For several reasons, including the fact that randomized trials are typically of short duration, cuas often combine data taken from randomized trials with the use of decision analytic modelling. Similarly, the present analysis derived estimates of clinical effectiveness (dfs and os) and resource use from the mosaic trial and extrapolated the clinical benefits and costs specific to each regimen over the lifetime (50 years) of the patient cohort.

The primary outcome measure chosen for this economic evaluation was the qaly; however, disease-free years (dfys) and lys gained are also reported. The qaly reflects os, adjusted by health-related quality of life. To calculate qalys, life-years are multiplied by utility values that represent preference-based values for health states. Utility values vary depending on age, sex, and the occurrence of relapse or adverse events. Because utility values were not collected in the mosaic trial, these estimates were derived from the available literature.

The analysis included disease-related health care costs from the perspective of the Ontario Ministry of Health and Long-Term Care. Resource utilization data were derived from the mosaic trial, and unit costs were derived from the Ontario Ministry of Health and Long-Term Care, the London Health Sciences Centre, and the published literature. Costs and outcomes were discounted to present values at a rate of 5% per year 9.

To test the robustness of the base-case results to variations in input parameters and assumptions, one-way sensitivity analyses were performed on discount rates, utility values, the choice of time horizon, and patient population (stages ii and iii).

### 2.2 Patient Population

The inclusion criteria for patients in the mosaic trial have been published 3. To summarize, patients were between 18 and 75 years of age and had undergone complete surgical resection of histologically proven stage ii (T3 or T4, N0, M0) or stage iii (any T, N1 or N2, M0) colon cancer. For our base case analysis, only patients with stage iii colon cancer were considered.

### 2.3 Treatment Regimens

Treatment commenced no later than 7 weeks post surgery. Patients were randomized to these treatment groups:

- 5fu/lv group: (given on both days 1 and 2 every 14 days for 12 cycles) a 2-hour infusion of lv 200 mg/m^2^, followed by a bolus of 5fu 400 mg/m^2^, followed by a 22-hour protracted infusion of 5fu 600 mg/m^2^
- folfox group: exactly the same as for the 5fu/lv group, except that a 2-hour infusion of oxaliplatin 85 mg/m2 is given simultaneously with the 2-hour infusion of lv 200 mg/m2 on day 1 only

### 2.4 Effectiveness Assessment

Treatment effectiveness was summarized in terms of qalys so as to capture survival as well as health-related quality of life.

#### 2.4.1 Survival

The primary endpoint of the mosaic trial was dfs. The median period of follow-up in that trial was 44.2 months at the time that the present economic evaluation was developed. Patient-level data regarding os was, therefore, derived directly from the mosaic trial for 4 years and was extrapolated to a lifetime horizon. The os for a full lifespan was derived by extrapolating the dfs trial data. The method of extrapolation has been described in full detail in Aballéa *et al.* 2007^10^. In brief, it was assumed that survival of patients in the first 4 years matched the experience of the participants in the mosaic trial. Survival beyond year 4 and to the end of year 5 was extrapolated using Weibull distributions, and survival beyond year 5 was assumed to match survival in the general population as observed in standard Canadian life tables 11. This final assumption required no recurrences of colon cancer beyond 5 years from diagnosis.

#### 2.4.2 Quality of Life and Health Utility

The cua uses the qaly as a composite measure that combines the length of a patient’s life with the quality of life that the patient experiences, where quality of life is measured on a 0 to 1 health utility scale. For example, 5 years at perfect health (utility 1) is worth 5 qalys; 5 years in poor health (for example, utility 0.5) is worth 2.5 qalys. To conduct the cua, estimates of utility were required for disease-free patients with stage iii colon cancer, and for relapsed patients with stage iii colon cancer. Disutilities associated with chemotherapy toxicities were also incorporated into the analysis.

##### Utility Associated with “Disease-free” Stage III Colon Cancer

Based on the studies of van den Brink *et al.* 12 and Ness *et al.* 13, the utility values for stage iii colon cancer patients diagnosed with the disease or surviving disease-free were in the range 0.63–0.89. A midpoint value of 0.76 was chosen, meaning that each patient entered the model with a utility of 0.76. For patients who remained “disease-free,” annual utility from baseline to year 5 was adjusted to account for declining health as a consequence of aging.

Patients who remained disease-free for 5 years were considered “cured,” and their utility was set to that of the general population (specific to the age and sex of the patient).

##### Utility Associated with Relapsed Stage III Colon Cancer

Based again on the studies of van den Brink *et al.* 12 and Ness *et al.* 13, utility values for relapse were reported to be in the range 0.24–0.67. We therefore assumed a midpoint value of 0.45 in the base-case analysis for patients with relapsed colon cancer. The patient’s annual utility declined from that point forward, until death, to account for aging.

##### Utility Associated with Chemotherapy Toxicities

Finally, utility decrements were incorporated into the model for the proportion of patients experiencing the following types of chemotherapy-related toxicities: grades 3 and 4 neutropenia, grades 2–4 neuropathy, grades 2–4 nausea and vomiting, and grades 2–4 diarrhea. The proportion of patients experiencing these toxicities was derived from the mosaic study. Estimates of utility associated with chemotherapy-related toxicities in colon cancer were not available in the literature. Accordingly, utility reductions associated with adverse events occurring following chemotherapies for other cancers were extracted from the literature. The utility decrements used in our model were previously detailed by Aballéa *et al.* 10 in the U.S. cost-effectiveness analysis.

### 2.5 Costs and Resource Utilization

Resource utilization data were derived from the mosaic trial, supplemented with data from the literature and validated using expert opinion at the Ottawa Hospital Regional Cancer Centre. Unit costs were derived from the London Health Sciences Centre, the Ontario Ministry of Health and Long-Term Care, relevant Web sites such as the cco site, and the published literature. The types of costs included were study chemotherapy, pre-treatment medications, replacement chemotherapy in the presence of toxicities, serious and non-serious adverse events, disease and toxicity within the trial, and costs of relapses. Costs are reported in 2006 Canadian dollars (CA$).

#### 2.5.1 Costs of Study Chemotherapy

2.5.1

The costs associated with study chemotherapy included the costs of the 6-month course of treatment with either folfox or 5fu/lv. These costs were calculated using the doses actually administered in the mosaic trial. The cost for 5fu/lv was taken from the cco Drug Formulary in November 2006^8^, which reported that cost to be CA$19 per cycle. Oxaliplatin is used at a dose of 85 mg/m^2^, and thus, for a patient with a body surface area of 1.75 m^2^, the cost per cycle of oxaliplatin used in the model was CA$1487^14^.

In addition to drug acquisition costs, “study chemotherapy costs” included the costs associated with drug administration, including inpatient or outpatient visit, infusion pump, insertion of a peripheral catheter line, pharmacy costs, and nursing costs. The only difference in costs between the two arms was the extra time required to administer the oxaliplatin, and a slightly higher estimate of the pharmacy fee.

#### 2.5.2 Costs of Pre-Treatment Medications

In addition to study chemotherapy, patients are often provided with prophylactic medications for chemotherapy-induced side effects. It was assumed that patients received oral ondansetron 8 mg twice daily for 3 days and intravenous dexamethasone (8 mg/mL).

#### 2.5.3 Costs of Adverse Events

For each serious adverse event reported in the trial, the corresponding costs (based on codes in the *International Statistical Classification of Diseases and Related Health Problems,* 10th revision 15) were collected from a data abstraction through the London Health Sciences Centre. Table I outlines the serious adverse event costs incorporated into the model. Table II outlines the costs of non-serious adverse events. The folfox regimen was associated with higher levels of adverse events than were seen with 5fu/lv, including significant differences in neutropenia (with and without fever or infection), thrombocytopenia, paresthesia, nausea and vomiting, diarrhea, and allergic reactions 3.

#### 2.5.4 Costs of Disease and Toxicity Monitoring Within the Trial

The costs of disease monitoring included outpatient visits such as follow-up visits, laboratory tests (for example, platelets, hemoglobin, neutrophils, bilirubin, serum glutamic oxaloacetic transaminase, serum glutamic pyruvic transaminase, alkaline phosphatase, carcinoembryonic antigens) and radiologic evaluations (for example, chest radiography, abdominal ultrasonography, abdominopelvic computerized tomography) and colonoscopies.

#### 2.5.5 Costs of Relapse

The treatment of relapse was assumed to be independent of the study treatment arm. A 2003 study by Maroun *et al.* 16 estimated the direct health care costs associated with the lifetime management of patients with a diagnosis of colon and rectal cancer in Canada. Of the 487 patients that relapsed in the mosaic trial, 19% had local recurrences and 81% had metastatic recurrences.

### 2.6 Sensitivity Analysis

Our base-case analysis compared the folfox regimen with 5fu/lv in stage iii colon cancer patients over a lifetime horizon. To assess the robustness of our results to changes in input parameters and assumptions, one-way sensitivity analyses were run according to these scenarios:

- Utility values associated with chemotherapy-related toxicities were varied by plus or minus 20%.
- Utility values associated with relapse were altered to 0, 0.24, and 0.67 (the base-case assumption was 0.45).
- Utility values associated with hospitalization were varied from 0% to 100% decrement when the base case was set at a 50% decrement.

A sensitivity analysis was also conducted that considered the full patient population of the mosaic study, including both stage ii and stage iii patients. Finally, a sensitivity analysis was conducted that considered a 4-year time horizon (the duration of follow-up in the mosaic study).

## 3. RESULTS

### 3.1 Effectiveness

**FIGURE 1:**
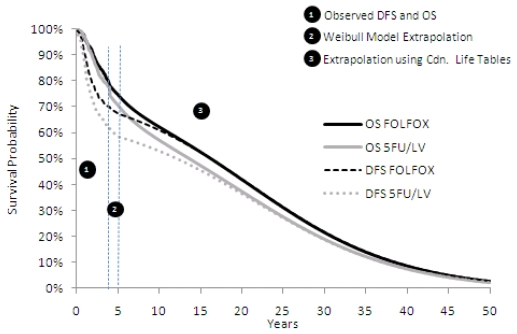
Within-trial and extrapolated survival differences over time. OS = overall survival; DFS=disease-free survival; FOLFOX = folinic acid, 5-fluorouracil, oxaliplatin; 5FU/LV = leucovorin/5-fluorouracil

Figure 1 summarizes the dfs and os differences between the two regimens at 4 years (within-trial), between 4 and 5 years, and extrapolated to 50 years.

During the initial 4-year follow-up, 194 patients treated with folfox either relapsed or died, compared with 245 patients treated with 5fu/lv alone 17. The Kaplan–Meier analysis of the mosaic trial predicted that 69.1% of patients in the oxaliplatin arm would be disease-free at 4 years compared with 60.7% of patients in the 5fu/lv arm (log-rank test: *p* = 0.002). Within-trial dfs and os translated into gains, with discounting, of 0.192 dfys and 0.054 lys for folfox-treated patients, as shown in Table III.

By using a Weibull extrapolation to fit the model to the tail end of the within-trial survival curve, dfs and os were extrapolated from 4 years to 5 years (60 months). The gains observed between the 4-year and 5-year time periods were added to the improvements observed up to 4 years, resulting in differences in favour of oxaliplatin of 0.262 and 0.085 in dfys and lys respectively.

Using Canadian life tables to extrapolate the os and dfs curves, with discounting, total accruals for folfox-treated patients were 1.08 and 0.653 in dfys and lys respectively.

### 3.2 Cost Outcomes

As illustrated in Table IV, the largest contributor to total cost for each treatment arm was chemotherapy, at CA$15,665 for folfox and CA$1757 for 5fu/lv (incremental difference: CA$13,908). The cost of the chemotherapy was based on the actual chemotherapy administered in the mosaic trial. The planned 12 cycles of adjuvant therapy were received by 86% of patients in the 5fu/lv group and 75% of patients in the folfox group. The median relative dose intensity of 5fu received was 84% for the folfox group and 98% for the 5fu/lv group. Costs associated with relapse on treatment and relapse during follow-up were lower with the folfox regimen. However, higher drug acquisition costs for folfox and costs associated with treating chemotherapy-related toxicities were major cost drivers, resulting in an incremental cost of CA$15,409 for folfox patients over a lifetime horizon.

### 3.3 Cost-Effectiveness

The base-case analysis demonstrated that adjuvant chemotherapy was more effective with folfox than with 5fu/lv, but it was also more costly. The lys and qalys were higher because of greater dfs and os in the folfox arm as compared with the arm using 5fu/lv alone. Table V shows cost per ly saved and cost per qaly gained for folfox as compared with 5fu/lv.

### 3.4 Sensitivity Analyses

A number of sensitivity analyses were conducted to evaluate the robustness of the base-case results. Most of the sensitivity analyses had little effect on the results. Interestingly, including stage ii patients from the mosaic trial still resulted in a cost per qaly of CA$33,534 over a lifetime horizon. However, as expected, limiting the analysis to 4 years resulted in a cost-effectiveness ratio for stage iii patients of more than CA$200,000 per qaly gained.

There are concerns with the external validity of the utility estimates, but the estimates used in the base-case analysis represent the best available data. Uncertainty in connection with the utility decrement associated with relapse had a large impact on the results of the within-trial analysis (for example, the 4-year time horizon); however, the effect of this parameter on the long-term results is modest. The incremental cost-effectiveness ratio (icer) was CA$30,402 when no utility decrement associated with relapse was assumed; that figure decreased to CA$21,138 when a 0.24 utility for a relapse was estimated.

## 4. DISCUSSION

After a complete surgical resection (undertaken with curative intent), patients with stage iii colon cancer have a 50%–60% chance of developing recurrent disease 18. New therapies that improve dfs in these patients, such as folfox, are thus vital. Based on the mosaic study, we therefore analyzed the cost-effectiveness of the folfox regimen in stage iii patients.

Cost-effectiveness analysis using qalys permits funding agencies to consider the value of alternative therapies, taking into account costs in addition to both quantity and quality of survival. Although patients on folfox had a greater incidence of toxicities than did those on 5fu/lv, the negative effect of the toxicities on qalys was outweighed by the qalys gained from improved survival with folfox. The gain in qalys was obtained at a reasonable cost-per-qaly ratio of CA$24,104.

The results from our evaluation appear to be very similar to the U.S. cost-effectiveness results presented by Aballéa *et al.* 10 After discounting costs and outcomes at 3% per annum, the U.S. evaluation reported an icer of US$20,600 per ly gained and US$22,800 per qaly gained. Likewise, the analysis submitted to the National Institute for Clinical Excellence by Sanofi–Aventis U.K. presented a cost–utility ratio of £4805 per qaly gained over a lifetime horizon 18. Comparing the qaly estimates, the Canadian and the U.K. models were very similar, with incremental differences between the two arms of 0.68 in the U.K. model and 0.639 in the Canadian model. The differences resulted from the use of different discount rates in the two analyses and also from some updated utility estimates that were available for the Canadian analysis. The results were higher in Canada and the United States as compared with the United Kingdom because of the relative difference in the price of oxaliplatin as compared with 5fu/lv between the countries. In the same disease setting, the estimated cost per ly gained for capecitabine as compared with 5fu/lv was £3899 (after discounting at 3.5% for costs and outcomes) 19.

A limitation of the mosaic trial data in informing this economic analysis is that mosaic was not designed for such purposes and was not powered to detect a significant difference in os. The dfs data from the mosaic trial was used to calculate recurrence rates—and ultimately to estimate survival. Thus, the value of dfs as a predictor of longer-term os is key to this analysis. The literature suggests that this predictive power holds, because a meta-analysis of clinical trials on adjuvant colon cancer has shown that dfs at 3 years is a strong predictor of os at 5 years 20. Moreover, since the time of the original evaluation, the mosaic trial investigators have published 6-year os data 5. We were thus able to test the predictive validity of the model at the 6-year time point. For stage iii patients, the os reported was 72.9% for folfox and 68.7% for 5fu/lv 5. The model predicted 71.4% for folfox and 67.1% for 5fu/lv. These results illustrate that, at the 6-year point, the model was within 1.5% of the actual os observed in the trial.

Another limitation is that many of the resource utilization estimates were informed from the trial. Should some of the resource utilization have been protocol-driven, then resource utilization in our analysis may have been overestimated. In an attempt to correct for this possibility, we held consultations with clinicians to ensure that assumptions in our analysis reflected current practice.

Finally, the availability of relevant utility values for the calculation of qalys was limited. We believe that the evaluation presented here used the best available data; the results of the sensitivity analysis suggest that the effect of this parameter on long-term results is modest.

Based in part on the information presented in the present economic evaluation, the province of Ontario recommended that oxaliplatin in combination with 5fu/lv (folfox) be funded for the adjuvant treatment of colon cancer. Based on the biologic similarities between colon and rectal cancer, the provinces of Ontario and British Columbia have both extended their funding to include rectal cancer. Within the clinical community, the strong evidence put forward by the Intergroup N9741 study 21 and by the mosaic trial have led to oxaliplatin being part of the standard of care of colorectal cancer in Canada.

## 5. CONCLUSIONS

The analysis presented here demonstrates that adding oxaliplatin to 5fu/lv in the adjuvant setting in patients with stage iii colon cancer represents a cost-effective use of resources.

## Figures and Tables

**TABLE I:** Costs of serious adverse events

icd-10 code	Description	Length of stay (days)	Cost per case (2006 CA$)
R50	Fever	4.02	4,304
T88.7	Allergic reaction	1	1,438
K52.9	Diarrhea	7.95	8,355
J13 J18.9	Pneumonia	7.97	7,581
D70	Neutropenic sepsis	6.26	7,285
R11.1, R11.2, R11.3	Nausea and vomiting	3.64	2,969
I80.2	Deep leg thrombophlebitis	9.00	6,754
078	Pulmonary embolism	6.30	6,676
R10	Abdominal pain	2.6	2,390
K56.6	Intestinal obstruction	9.7	11,529
T80	Injection site reaction	3.0	3,305

icd-10 = *International Statistical Classification of Diseases and Related Health Problems,* 10th revision 16.

**TABLE II:** Costs of non-serious adverse events

Description	Treatment assumption	Cost per case (2006 CA$)
Nausea and vomiting		10.76/episode
Grade 2	Prochlorperazine (10 mg) in combination with oral dexamethasone (4 mg), 4 times daily for 3 days	
Grades 3 and 4	Intravenous prochlorperazine and oral dexamethasone (8 mg) for 3 days	13.12/episode
Neutropenia		
Grade 2	No treatment	0
Grades 3 and 4	Admitted to hospital	5197
Diarrhea	Loperamide (16 mg daily for 12 days); stool culture and test for *Clostridium difficile*	25.75

**TABLE III:** Incremental health outcomes, discounted by 5%

Time horizon	Outcome	folfox	5fu/lv	Difference
4 Years	dfys	3.042	2.850	0.192
	lys	3.372	3.318	0.054
	qalys	2.440	2.373	0.067
5 Years	dfys	3.605	3.343	0.262
	lys	4.003	3.917	0.085
	qalys	NA	NA	NA
50 Years	dfys	9.861	8.781	1.080
	lys	10.418	9.765	0.653
	qalys	8.048	7.409	0.639

folfox = folinic acid, 5-fluorouracil, oxaliplatin; 5fu = 5-fluorouracil; lv = leucovorin; dfys = disease-free years; lys = life-years; qalys = quality-adjusted life-years; na = not available.

**TABLE IV:** Disaggregated within-trial and beyond-trial costs in 2006 Canadian dollars, discounted by 5%

Cost category	Costs		
	folfox	5fu/lv	Incremental
Within-trial costs			
Study chemotherapy	15,665	1,757	13,908
Outpatient visits	638	661	−23
Lab tests	236	244	−8
Radiologic evaluations	1,837	1,889	−52
Neutropenia	2,553	226	2,328
Neuropathy	0	0	0
Diarrhea	13	11	2
Nausea and vomiting	10	3	6
Serious adverse events	1,677	1,041	636
Relapse treatment	3,074	4,175	−1,100
Relapse follow-up	429	618	−189
TOTAL	26,133	10,623	15,509
Beyond-trial costs			
Relapse	200	297	−97
Follow-up	1,106	998	108
Other	287	398	−111
LIFETIME TOTAL	27,726	12,317	15,409

folfox = folinic acid, 5-fluorouracil, oxaliplatin; 5fu = 5-fluorouracil; lv = leucovorin.

**TABLE V:** Cost–utility analysis

	Costs (2006 CA$)	dfys	lys	qalys
Regimen				
folfox	27,726	9.861	10.418	8.048
5fu/lv	12,317	8.781	9.765	7.409
Incremental	15,409	1.080	0.653	0.639
icer^a^		14,266	23,598	24,104

dfy = disease-free years; ly = life-years; qaly = quality-adjusted life-years; folfox = folinic acid, 5-fluorouracil, oxaliplatin; 5fu = 5-fluorouracil; lv = leucovorin; icer = incremental costeffectiveness ratio.
